# A Microfluidic Chip Using Phenol Formaldehyde Resin for Uniform-Sized Polycaprolactone and Chitosan Microparticle Generation

**DOI:** 10.3390/molecules18066521

**Published:** 2013-06-03

**Authors:** Yung-Sheng Lin, Chih-Hui Yang, Chin-Tung Wu, Alexandru Mihai Grumezescu, Chih-Yu Wang, Wan-Chen Hsieh, Szu-Yu Chen, Keng-Shiang Huang

**Affiliations:** 1 Department of Applied Cosmetology and Master Program of Cosmetic Science, Hungkuang University, Taichung 43302, Taiwan; E-Mail: linys@sunrise.hk.edu.tw; 2 Department of Biological Science and Technology, I-Shou University, Kaohsiung 82445, Taiwan; E-Mails: chyang@isu.edu.tw (C.-H.Y.); wanjenxie@gmail.com (W.-C.H.); isu9941008a@cloud.isu.edu.tw (S.-Y.C.); 3 Department of Computer Science and Information Engineering, Providence University, Taichung 43301, Taiwan; E-Mail: nxx367@yahoo.com.tw; 4 Department of Science and Engineering of Oxidic Materials and Nanomaterials, Faculty of Applied Chemistry and Materials Science, University Politehnica of Bucharest, Bucharest 011061, Romania; E-Mail: grumezescu@yahoo.com; 5 Department of Biomedical Engineering, I-Shou University, Kaohsiung 82445, Taiwan; E-Mail: crab@isu.edu.tw; 6 The School of Chinese Medicine for Post-Baccalaureate, I-Shou University, Kaohsiung 82445, Taiwan

**Keywords:** microfluidics, emulsion, droplet, microparticles, phenol formaldehyde resin, polycaprolactone, chitosan

## Abstract

This study develops a new solvent-compatible microfluidic chip based on phenol formaldehyde resin (PFR). In addition to its solvent-resistant characteristics, this microfluidic platform also features easy fabrication, organization, decomposition for cleaning, and reusability compared with conventional chips. Both solvent-dependent (e.g., polycaprolactone) and nonsolvent-dependent (e.g., chitosan) microparticles were successfully prepared. The size of emulsion droplets could be easily adjusted by tuning the flow rates of the dispersed/continuous phases. After evaporation, polycaprolactone microparticles ranging from 29.3 to 62.7 μm and chitosan microparticles ranging from 215.5 to 566.3 μm were obtained with a 10% relative standard deviation in size. The proposed PFR microfluidic platform has the advantages of active control of the particle size with a narrow size distribution as well as a simple and low cost process with a high throughput.

## 1. Introduction

Microfluidics is a set of technologies for manipulating nanoliter volumes of fluids in channels with dimensions measured in tenths or even hundreds of micrometers [1]. In the early periods of microfluidic technology in the 1990s, most microfluidic chips were made of silicon and glass [2,3]. Although these materials have good surface qualities and precise properties, there remain some inconveniences in their applications. Silicone or glass/quartz-based products are usually expensive and have low impact strength [4]. Silicone or glass-based fabrication processes are labor intensive and time-consuming, and require costly clean-room facilities and instruments for their photolithography and etching processes, so from a material viewpoint, silicon or glass/quartz-based microfluidic chips have some limitations in practice.

Other rigid materials such as steel or aluminum provide alternative choices for a microfluidic chip substrate [5]. They have advantages such as non-permeable walls, good heat conductivity, robust stability, and durable operation under harsh reaction conditions such as high temperature and/or strong organic solvent systems for certain applications. The limitations of those materials for microfluidic devices are that they are difficult, time-consuming, and costly to fabricate [6].

Poly(dimethylsiloxane) (PDMS) is a soft material and has rapidly become an alternative for microfluidic chips. This material possesses many attractive properties for microfluidics. The major advantages are its easy micromolding fabrication and elastomeric properties. The nontoxic nature and gas permeability also allow its biomedical use. However, its applications suffer from serious swelling and PDMS oligomer leaching in many organic solvents. The limited compatibility of PDMS material with various organic solvents was reported in detail by Whitesides’s group [7].

The alternative material for PDMS focuses on the fluorocarbon-based polymer characterized by a high resistance to organic solvents [8,9,10]. Among them, polytetrafluoroethylene (PTFE) has been widely applied for various microdevices [11,12,13]. Compared with common commercial polymers, the cost of PTFE is high. This price downside causes several restrictions on its applications.

Introduced in 1909, phenol formaldehyde resin (PFR) is discovered earlier than fluorocarbon polymer. PFR in the form of Bakelite was the earliest commercial synthetic resin. PFR is widely used as a commodity and engineered material in applications such as circuit boards, coatings, adhesives and molded products, including pool balls and laboratory countertops [14]. Recently, biocompatible and green luminescent monodispersed PFR nanoparticles for bioanalytical and biosensing applications have been reported [15]. The PFR material is cheap, solvent-resistant, and easily processed. Furthermore, it is readily available in Taiwan because of Taiwan’s important strengths in electronics manufacturing and its huge consumer electronics market in the World. However, nowadays few studies have applied the conventional PFR material for microfluidic chips.

Polycaprolactone (PCL) and chitosan microspheres have gained a great deal of attention for biomedical applications. These microspheres can be used as a means of enhancing drug delivery efficiency, biomedical diagnostics and target manipulation. PCL is non-toxic in nature and found to be cyto-compatible with a several body tissues, which makes it a perfect material for tissue engineering. PCL has flexible mechanical properties that are suitable for paramedical applications, wound dressings, and dentistry [16,17]. This substantiates the functionality of the polymer in pharmaceutical dosage form design and development. Chitosan, a natural biodegradable polymer, is of great interest in biomedical research due to its excellent properties including bioavailability, nontoxicity, high charge density, and mucoadhesivity, which creates an immense potential for various biomedical and pharmaceutical applications such as drug delivery, tissue engineering, cosmetics, and food and nutrition products [18,19,20,21]. Hydrophobic PCL and hydrophilic chitosan can be considered representative of solvent- and nonsolvent-dependent polymers, respectively. We use these two representative polymers to demonstrate the compatibility of the PFR chip with the organic solvent and water.

Based on our previous microfluidic platforms for the generation of various polymer particles [22,23,24,25,26,27,28,29,30,31], this study provides a new solvent-compatible microfluidic chip based on PFR to prepare uniform microparticles. This is the first report using the PFR microfluidic chip to generate both hydrophobic and hydrophilic material microparticles. The solvent-dependent polycaprolactone and nonsolvent-dependent chitosan were demonstrated to be applicable on the same platform. The proposed PFR microfluidic platform provides great promise for applications in particle generation.

## 2. Results and Discussion

Conventional microfluidic chips are difficult to clean properly because of the bonded sealing design [32,33,34,35]. To overcome this drawback, the proposed PFR microfluidic chip is designed to be disassembled for microchannel cleaning. Three layers connected by twenty M4 screws could be easily taken apart for further cleaning after each experiment.

**Figure 1:**
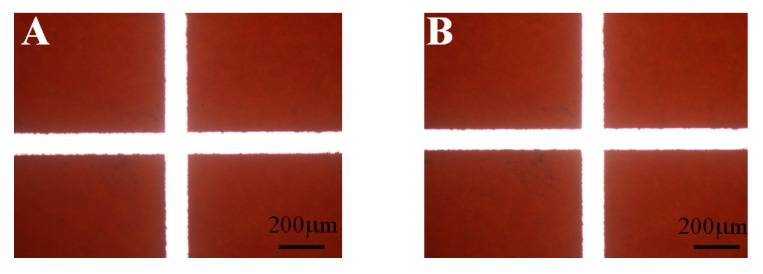
Appearance of the cross-junction microchannel in the PFR chip before (**A**) and after (**B**) contact with chloroform for 24 h.

Generally, phenol formaldehyde resin can dissolve in many organic solvents. The prepared PFR microfluidic chip thus has some limitations for a long contact with organic solvents. However, there were no problems in the synthesis of polymer microparticles using the PFR microfluidic chip for 24 h with chloroform in this study, as shown in Figure 1.

**Figure 2:**
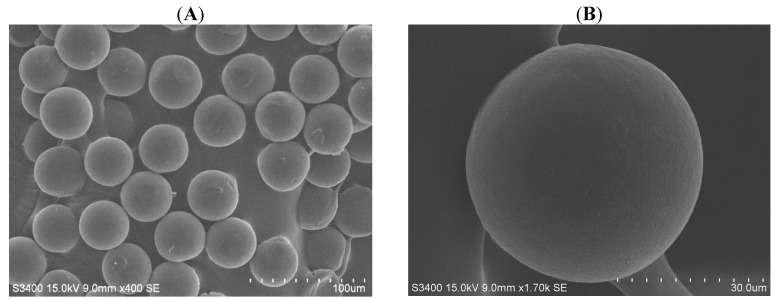
Pictures of PCL microparticles in scanning electron microscope. (**A**) magnification 400× and (**B**) magnification 7,000×.

Figure 2 shows PCL microparticles collected under the condition of a 0.3 mL/min continuous flow rate and a 0.2 mL/h dispersed flow rate. The scanning electron microscope (SEM) picture reveals that PCL microparticles were consistent in morphology, and exhibited excellent size uniformity (Figure 2A The average diameter of the PCL microparticles was 46.1 ± 3.1 µm. The zoom-in image (Figure 2B) shows that of the PCL microparticles had smooth surface and good sphericity. 

**Figure 3:**
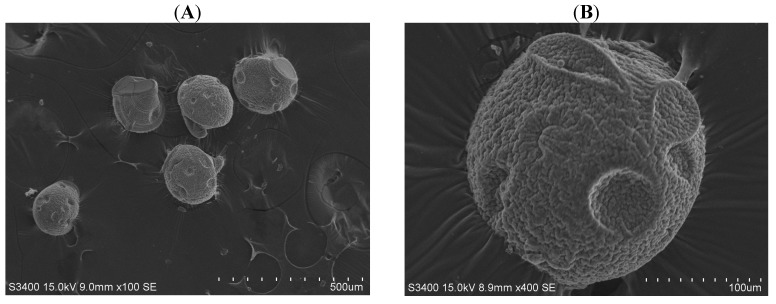
Pictures of chitosan microparticles in scanning electron microscope. (**A**) magnification 100× and (**B**) magnification 400×.

Figure 3 displays chitosan microparticles collected under the condition of a 0.5 mL/minute continuous flow rate and a 0.04 mL/hour dispersed flow rate. Compared with PCL microparticles, chitosan microparticles were less consistent in both morphology and size due to their less rigid properties (Figure 3A). In spite of the differences in chitosan microparticle sizes, the size of microparticles could still be controlled within about a 10% relative standard deviation (R.S.D., defined as the ratio of standard deviation to average). The average diameter of the chitosan microparticles was 215.5 ± 8.9 µm. The chitosan microparticle had some sunken regions and a wrinkled surface as shown in Figure 3B.

**Figure 4:**
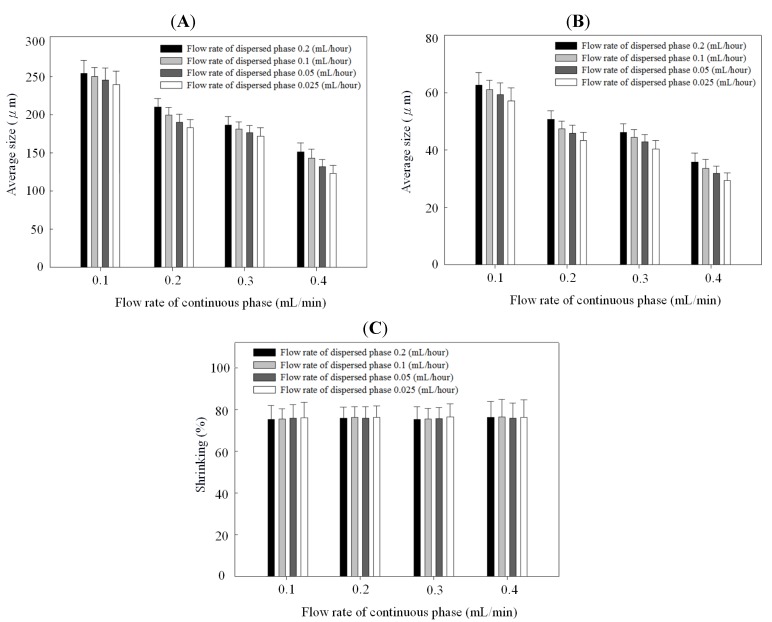
Droplet size (**A**), microparticle size (**B**), and shrinking ratio (**C**) of polycaprolactone in different continuous/dispersed flow rates.

**Figure 5:**
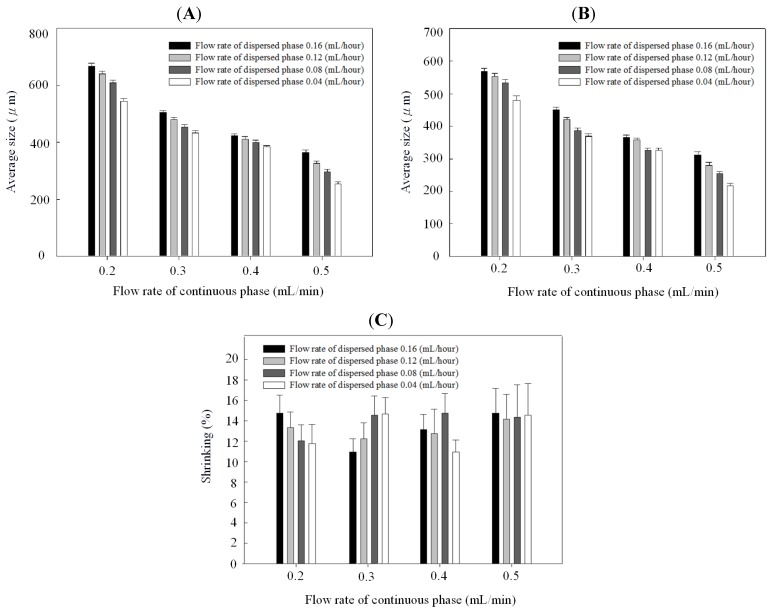
Droplet size (**A**), microparticle size (**B**), and shrinking ratio (**C**) of chitosan in different continuous/dispersed flow rates.

Figure 4 and Figure 5 show the relationships between droplet/microparticle sizes and continuous/dispersed flow rates for PCL and chitosan, respectively. The small relative standard deviation in size of particles including emulsion droplets and dry solid microparticles indicates the good uniformity of particles obtained in this microfluidic platform. This uniformity corresponds to the results of monodispersed microparticles in Figure 2A and Figure 3A. 

At the same concentration of polymer solution (1% wt/v), both emulsion droplets and dry solid microparticles of PCL were smaller than chitosan. The emulsion droplet size with different materials (polycaprolactone and chitosan) varied significantly, resulting from the different competition between viscous stresses and capillary stresses between the two phases. Furthermore, the shrinking ratio (from a droplet to a dry microparticle) of PCL was higher than for chitosan, probably due to a more dense structure in the PCL microparticles. In the solvent evaporation process, polycaprolactone molecules rearranged and packed to condense the emulsion droplet to be the dry microparticle. There was a high shrinkage ratio in this solidification process. However, chitosan molecules underwent an extra crosslinking to form a gel in the emulsion droplet. Compared with polycaprolactone, the crosslinked chitosan molecules had lower mobility for rearrangement and packing in the evaporation process, resulting in a low shrinkage ratio. Therefore, polycaprolactone droplets have a higher shrinkage ratio than chitosan.

Figure 4B and Figure 5B indicate that particles decreased with the continuous phase flow rate but increased with the dispersed phase flow rate. Effects of continuous/dispersed flow rates on the particle size agree with previous reports [22,23,24,25,26,27,28,29,30,31]. A higher continuous flow rate provided a greater shear force to accelerate the detachment of emulsion droplets from the dispersed phase flow. The shorter droplet formation time of the dispersed phase flow resulted in a smaller emulsion droplet in size. The 0.1–0.4 mL/min flow rate of PVA solution in PCL system and 0.2–0.5 mL/min of sunflower seed oil in chitosan system show the smaller particle size at a higher continuous flow rate. 

On the contrary, a higher dispersed flow rate increased the emulsion droplet size. Under the same droplet formation mechanism, the droplet volume increased with the dispersed flow rate due to the accumulated volume in a droplet. Consequently the size of particles is larger in a higher dispersed flow rate. The flow rate of 0.025~0.2 mL/h for PCL solution and 0.04–0.16 mL/h for chitosan solution show the larger particle size at a higher dispersed flow rate.

Different from droplet formation at the cross-junction in the microchannel [22,23,24,25,26,27,28,29,30,31], we used the jetting flow with a long neck to adjust the shear force breaking up the immiscible polymer solution into big emulsion droplets. Although the neck was long, it was still stable enough for regular emulsion droplet formation. This result agreed with the classic literature concerning liquid/liquid jet dispersion [36,37,38,39,40,41]. As shown in the SEM pictures, polycaprolactone and chitosan microparticles obtained from the jetting flow were uniform in both morphology and size.

## 3. Experimental

### 3.1. Materials

Poly(vinyl alcohol) (PVA, 88%–89% hydrolyzed), polycaprolactone (PCL), and chitosan were purchased from Sigma (Sigma Chemical Co., St. Louis, MO, USA). Sunflower seed oil was obtained from Uni-President Enterprises Corp., Tainan, Taiwan. Distilled water was filtered by a 0.22 nm filter (Millipore Inc., Clifton, NJ, USA) before use. All reagents were used as purchased without any further purification.

### 3.2. Construction of the Microfluidic Platform

**Figure 6:**
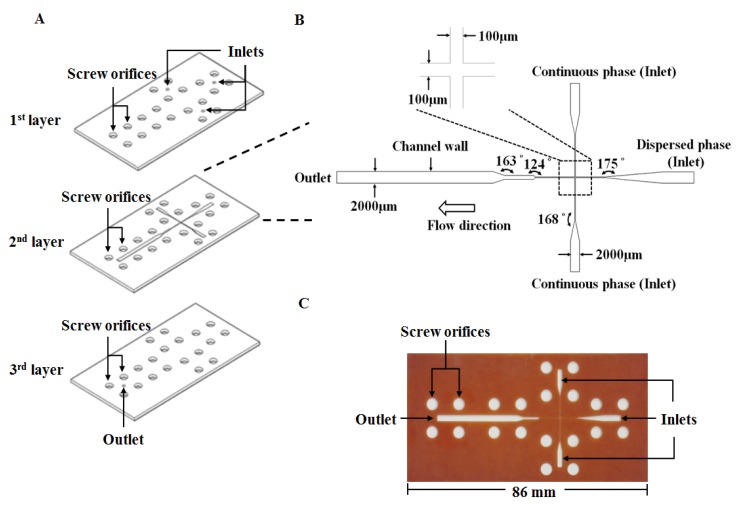
The proposed phenol formaldehyde resin-based microfluidic device: (**A**) The chip device in expanded view. (**B**) Geometry of the microfluidic channel. (**C**) A photograph of the microfluidic chip.

The proposed microfluidic chip was laid out on a conventional PFR substrate (length/width/depth: 270 mm/210 mm/1 mm). The PFR microfluidic chip was fabricated using an engraving micromachining process by an engraving machine (Twinsoft, Taipei, Taiwan). The microfluidic device (Figure 6A) consists of three layers which are from top to bottom: the cover layer (containing three inlets and twenty screw orifices for binding), the main layer (containing main microfluidic channel and screw orifices) and the bottom layer (containing one outlet and twenty screw orifices for binding), respectively. These three layers were integrated by twenty M4 screws (0.5 mm pitch, 4 mm diameter) to be the microfluidic device for preparing microparticles. Figure 6B shows the geometric dimension of the microchannel with a 100 μm wide cross-junction. The downstream of the cross-junction was designed to be gradually broadened to 2000 μm in width for slowing down the flow and enhancing observation of microdroplets. Figure 6C displays a photograph of the PFR microfluidic chip. 

### 3.3. Experimental Procedure

**Figure 7:**
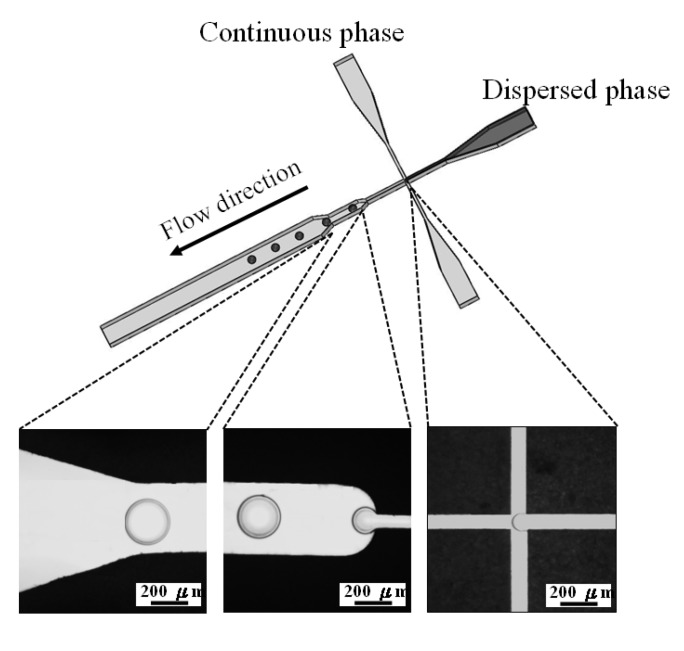
Schematic drawing of droplet formation in the cross-junction microchannel. The flow-focusing system generates uniform self-assembling droplets at the expansion site of the main channel.

Polycaprolactone and chitosan droplets were obtained by the emulsion process. The pair of dispersed and continuous phases for these two materials were polycaprolactone solution (1% wt/v in chloroform) and PVA (1% wt/v in distilled water), and chitosan solution (1% wt/v in 2% acetic acid) and sunflower seed oil, respectively. Two sets of independently controlled syringe pumps (Kd Scientific KDS101, Holliston, MA, USA) were used to separately inject dispersed and continuous phases into the microfluidic chip together. The dispersed phase was injected into the central channel, while the continuous phase was injected from the two side channels (Figure 7) hydrodynamically focusing a stream of polymers solution enabled the construction of polymer emulsions along the microchannel axis. The shear force at the downstream expansion site of cross-junction stretched dispersed phase into a droplet periodically as shown in the picture of Figure 7. These collected droplets further made process of evaporation and solidification in a 50 mL beaker at 37 °C for 24 h. All droplets were lyophilized for another 24 h to complete liquid removal to become dry solid microparticles. 

An optical microscope (TE2000U, Nikon, Lewisville, TX, USA) and scanning electron microscope (S-2700, Hitachi, Tokyo, Japan) were used to observe emulsion droplets and dry solid microparticles, respectively. To ensure statistical representation, one hundred particles including emulsion droplets and dry solid microparticles were counted to calculate the average particle size. The diameter of the particles was expressed as mean ± standard deviation.

## 4. Conclusions

This study developed a new microfluidic platform for uniform microparticle preparation. The phenol formaldehyde resin-based microfluidic chip is solvent-resistant and can be disassembled for cleaning to be reused. Prepared polycaprolactone and chitosan microparticles were uniform in size, and their sizes could be adjusted by changing the flow rates of the dispersed/continuous phases. Polycaprolactone microparticles had a higher shrinkage ratio than chitosan ones during the evaporation process. The proposed microfluidic platform is low cost, easy to organize and offers high throughput for uniform microparticle generation.
